# Increasing atmospheric CO_2_ and canopy temperature induces anatomical and physiological changes in leaves of the C_4_ forage species *Panicum maximum*

**DOI:** 10.1371/journal.pone.0212506

**Published:** 2019-02-19

**Authors:** Eduardo Habermann, Juca Abramo Barrera San Martin, Daniele Ribeiro Contin, Vitor Potenza Bossan, Anelize Barboza, Marcia Regina Braga, Milton Groppo, Carlos Alberto Martinez

**Affiliations:** 1 Department of Biology, FFCLRP, University of São Paulo, Ribeirão Preto, São Paulo, Brazil; 2 Department of Plant Physiology and Biochemistry, Institute of Botany, São Paulo, São Paulo, Brazil; Universidade Federal de Vicosa, BRAZIL

## Abstract

Changes in leaf anatomy and ultrastructure are associated with physiological performance in the context of plant adaptations to climate change. In this study, we investigated the isolated and combined effects of elevated atmospheric CO_2_ concentration ([CO_2_]) up to 600 μmol mol^-1^ (*eC*) and elevated temperature (*eT*) to 2°C more than the ambient canopy temperature on the ultrastructure, leaf anatomy, and physiology of *Panicum maximum* Jacq. grown under field conditions using combined free-air carbon dioxide enrichment (FACE) and temperature free-air controlled enhancement (T-FACE) systems. Plants grown under *eC* showed reduced stomatal density, stomatal index, stomatal conductance (*g*_s_), and leaf transpiration rate (*E*), increased soil-water content (*SWC*) conservation and adaxial epidermis thickness were also observed. The net photosynthesis rate (*A*) and intrinsic water-use efficiency (*iWUE*) were enhanced by 25% and 71%, respectively, with a concomitant increase in the size of starch grains in bundle sheath cells. Under air warming, we observed an increase in the thickness of the adaxial cuticle and a decrease in the leaf thickness, size of vascular bundles and bulliform cells, and starch content. Under *eCeT*, air warming offset the *eC* effects on *SWC* and *E*, and no interactions between [CO_2_] and temperature for leaf anatomy were observed. Elevated [CO_2_] exerted more effects on external characteristics, such as the epidermis anatomy and leaf gas exchange, while air warming affected mainly the leaf structure. We conclude that differential anatomical and physiological adjustments contributed to the acclimation of *P*. *maximum* growing under elevated [CO_2_] and air warming, improving the leaf biomass production under these conditions.

## Introduction

In the last five decades, human activities have resulted in the increased emission of greenhouse gases (GHGs) as atmospheric CO_2_ concentration ([CO_2_]) from approximately 320 μmol mol^-1^ to more than 400 μmol mol^-1^ [1]. This increase in GHGs is responsible for the rise in the global surface temperature [1]. The Intergovernmental Panel on Climate Change (IPCC) models indicated that the global mean temperature anomaly could exceed 2°C in 2100 [1], potentially affecting tropical ecosystems, such as perennial pastures [2, 3]. Pastures are the greatest feeding source for livestock, and future food security will depend on how forage species respond to global change variables [1]. Therefore, the acclimation mechanisms of tropical grasslands to increased atmospheric [CO_2_] and air warming have attracted attention recently.

Plant survival under rapid climate change occurs mainly due to phenotypic plasticity, with complex adjustments in plant physiology and the structure of leaf tissues [4]. Plant physiology, productivity, and leaf anatomy are closely linked because mesophyll characteristics affect carbon assimilation rates and leaf function [5]. Furthermore, the leaf tissue thickness is important in many processes, such as leaf thermal regulation, light interception, and CO_2_ and water vapor diffusion [6]. For forage species, the proportion of leaf tissues is essential in the digestibility and nutritional value for animals [7]. However, leaf anatomy is often neglected in studies that evaluate the plant responses to climate change variables [5].

In tropical and subtropical pastures, rainfed C4 grasses represent the main feeding source for cattle. Although it is important for livestock production, little is known about C4 pasture strategies for acclimation to elevated atmospheric [CO_2_] and air warming [8]. C3 and C4 species have distinct anatomical and physiological acclimation mechanisms under elevated [CO_2_] and temperature [9–11]. For a long time, the responses of C4 species to elevated [CO_2_] were considered marginal or inexistent due to the inherent-CO_2_ concentration mechanism in the photosynthesis process of C4 plants [12]. However, it is now known that C4 species respond to the increase in atmospheric [CO_2_] both directly and indirectly [10]. Different from C3 plants, C4 leaves growing under elevated [CO_2_] often show a reduced thickness of tissues, such as the epidermis and mesophyll, resulting in a decrease in the total leaf thickness [9, 13]. Cuticle deposition is often intensified, and the stomatal density may change, with a diversity of responses among species [10, 13, 14]. However, a reduced stomatal aperture is a typical response found in C4 plants [10]. Enhancements in the photosynthetic performance accompany all these changes in plants [10].

Different from the [CO_2_] effects, warmed C4 leaves often show an increase in the total leaf thickness [9]; however, exceptions are found [15]. Under air warming, the leaf surface shows a thicker cuticle, and an increased stomatal density, size, and conductance is often observed [15–17]. A photosynthetic response to the elevated temperature significantly depends on the optimum growth temperature of species, but is frequently enhanced in well-watered C4 plants [11]. However, the combined effects of elevated [CO_2_] and air warming on the leaf anatomy and physiology are scarce in the literature, especially for C_4_ species.

When different environmental factors are combined, interactive effects may occur, bringing new and unexpected responses [18]. Furthermore, most of our knowledge of plant responses to global change variables comes from experiments conducted on plots in open-top chambers, greenhouses, or other artificial conditions. These controlled environments may exacerbate the plant responses to abiotic stress and may not reflect the acclimation mechanisms that would be present under field conditions [10]. In this study, we used a combination of temperature free-air controlled enhancement (T-FACE) and free-air CO_2_ enrichment (FACE) technologies to increase the atmospheric [CO_2_] and canopy temperature under field conditions.

Brazil is the world’s second largest meat exporter and the fourth-most country in the world in terms of significant land area used in grazing systems [19]. *Panicum maximum* Jacq. (Synonym *Megathyrsus maximus* (Jacq.) B. K. Simon & S. W. L. Jacobs) (Poaceae, C4) cv. Mombaça is an important perennial grass used as pasture in tropical and subtropical regions, covering more than 7.7 million hectares in the Brazilian territory [20]. *P*. *maximum* has a Kranz anatomy (subtype PEP-CK), with an undifferentiated mesophyll with few intercellular spaces and one layer of concentric bundle sheath cells around vascular bundles, in which starch accumulates. The positioning of chloroplasts in the bundle sheath cells is centrifugal. The leaves are amphistomatic, and epidermis consists of a single layer of juxtaposed cells with bulliform cells inserted between the vascular bundles in the adaxial leaf surface. A previous study showed that *P*. *maximum* under a warmed, CO_2_-enriched atmosphere enhances its leaf expansion rates and aboveground biomass production [3]. However, the underlying anatomical and physiological acclimation mechanisms of *P*. *maximum* to future conditions of atmospheric [CO_2_] and air warming are not found in the literature.

Here, we aim to test the isolated and combined effects of elevated [CO_2_] and air warming on the ultrastructure, leaf anatomy, and physiology of *P*. *maximum* from an integrated perspective. We hypothesized that elevated [CO_2_] and air warming will differently change the structure of the leaf tissues and independently enhance the photosynthetic performance and biomass production of *P*. *maximum*.

## Material and methods

### Study site and system

The experiment was conducted during the 2015 Brazilian summer at the Trop-T-FACE facility, that combines free-air carbon dioxide enrichment (FACE) and temperature-free air enhancement (T-FACE) systems, located in Ribeirão Preto, São Paulo State, Brazil (21° 10′ 8′′ S, 47° 51′ 48.2′′ W). This region stands 580 m above sea level, with an annual precipitation of 1508 mm, an average annual temperature of 22°C, and soil classified as eutroferric red Oxisol of clayey texture.

Seeds *of Panicum maximum* cv. Mombaça were sowed in 16 plots of 10 × 10 m in fertilized soil with NPK 4-14-8 at a dose of 1 t ha^-1^ [21]. The planting density was 16 plant m^-2^ which is the common plant density for *P*. *maximum* cv Mombaça used by farmers [22]. Soil liming was performed 2 months before seeding to increase the soil pH from 4.5 to 5.5. During seedling growth, plants were under adequate conditions of water availability. After 2 months of growing and pasture establishment, when plants reached 90 cm height, plants were clipped at 30 cm above the ground (the usual practice from farmers), and the treatments with different levels of [CO_2_] and temperature were initiated. High leaf dry mass production and browsing efficacy of *P*. *maximum* cv Mombaça are achieved with 90 cm pre-grazing and 30 cm post-grazing pasture height targets, respectively [23]. Treatments were applied at the vegetative stage during 30 d of experiment with no irrigation other than rainfall. The 30-d experimental period is the normal plant regrowth time that is used in rotational grazing practices for this species [24]. It has been reported that *P*. *maximum* cv. Mombaça have rapid stem elongation, resulting in 95% canopy light interception at 90 cm [23]. We used a randomized four-block design and tested the effects of two levels of atmospheric [CO_2_]: ambient (*aC*) and elevated [CO_2_] (~600 μmol mol^-1^) (*eC*), and two levels of air temperature: ambient (*aT*) and elevated (+2°C more than canopy ambient temperature) (*eT*). Each block contained four different combinations: *aCaT* (ambient [CO_2_] and ambient temperature), *eCaT* (elevated [CO_2_] and ambient temperature), *aCeT* (ambient [CO_2_] and elevated temperature) and *eCeT* (elevated [CO_2_] and elevated temperature). Each circular plot consisted of a 2-m-diameter ring inside of 10 × 10 m plots, located 12 m away from each other to avoid CO_2_ contamination.

### Trop-T-FACE description

#### FACE system

To increase the atmospheric [CO_2_] by 600 μmol mol^-1^, we used a free-air CO_2_ enrichment system (FACE) [3, 25]. Briefly, PVC rings of 2 m diameter punctured with micro holes fumigated the plots with CO_2_. Through a proportional integration device algorithm (PID), the central unit regulated the amount of [CO_2_] needed in *eC* plots using [CO_2_] difference of *aC* and *eC* plots and wind speed data. A GMT222 CO_2_ transmitter sensor (Vaisala, Helsinki, Finland), installed in the center of each plot at canopy height, was used to monitor [CO_2_] during the experiment. Wind speed was monitored by an anemometer located in the center of the Trop-T-FACE facility at 3 m above the ground. Liquid CO_2_ used for fumigation was stored in a 12-ton cryogenic tank with a vaporizer unit. The target concentration of 600 μmol mol^-1^ was held from sunrise to sunset, and ‘dummy’ rings were installed in *aC* plots.

#### T-FACE system

We used a temperature free-air controlled enhancement system (T-FACE) [26] to increase the canopy temperature to +2°C more than the ambient canopy temperature (*eT*). In each *eT* plot, plants were warmed by six infrared Salamander heaters TFE 750–240 (Mor Electric Heating, Comstock Park, MI, USA) mounted on Salamander reflectors ALEX-F (Mor Electric Heating, Comstock Park, MI, USA) in a 2-m-diameter hexagonal pattern. The heaters were suspended 0.8 m above the canopy with aluminum bars and had their angle and height adjusted according to plant growth. T-FACE control temperature was performed by a PID control system installed in a CR1000 datalogger with AM25T multiplexors (Campbell Scientific, Logan, UT, USA) [27]. The control system integrates the canopy temperature of *aT* and *eT* plots and regulates the canopy temperature to 2°C more than the ambient canopy temperature in warmed plots. Each *eT* plot used an *aT* plot as a reference for temperature control of the same experimental block. The same aluminum structure and ‘dummy’ heaters were installed in *aT* plots. T-FACE worked continuously during the treatment period of the experiment. Trop-T-FACE data was monitored and collected during the treatment period of the experiment using Loggernet software (Campbell Scientific, Logan, UT, USA). We used an automatic microclimate station WS-PH1 connected to a data logger DL2e (Delta-T Devices, Cambridge, UK) to monitor and store the meteorological data: total solar radiation (*Rad*), air temperature (*T*_*air*_), and relative air humidity *(Rh*) during the whole growing season. Precipitation data was obtained from a weather station located near to the Trop-T-FACE facility. Soil temperature (*T*_*soil*_) was monitored hourly by ST2 sensors located in the center of each plot at 10 cm deep and connected to a data logger DL2e (Delta-T Devices Ltd., Burwell, Cambridge, UK).

### Meteorological conditions

High rainfall intensity occurred during the 30-d experimental period, with an accumulated precipitation level of 224 mm (S1A Fig). Days were usually cloud-free before midday and cloudy during the rest of the day. The daily average total solar radiation (*Rad*) was 0.33 kW m^-2^ (S1A Fig), and the maximum total solar radiation was 1.08 kW m^-2^ during the experiment. During the growing season, the average relative air humidity (*Rh*) was 87% (S1B Fig), with minimum values of 38%. The average air temperature (*T*_*air*)_ during the experimental period was 25°C (S1B Fig), and the maximum and minimum *T*_*air*_ values registered during the experiment were 35°C and 16°C, respectively. The soil temperature of *eT* plots was on average 0.7°C warmer than that under *aT* plots (S1C Fig).

**Fig 1:**
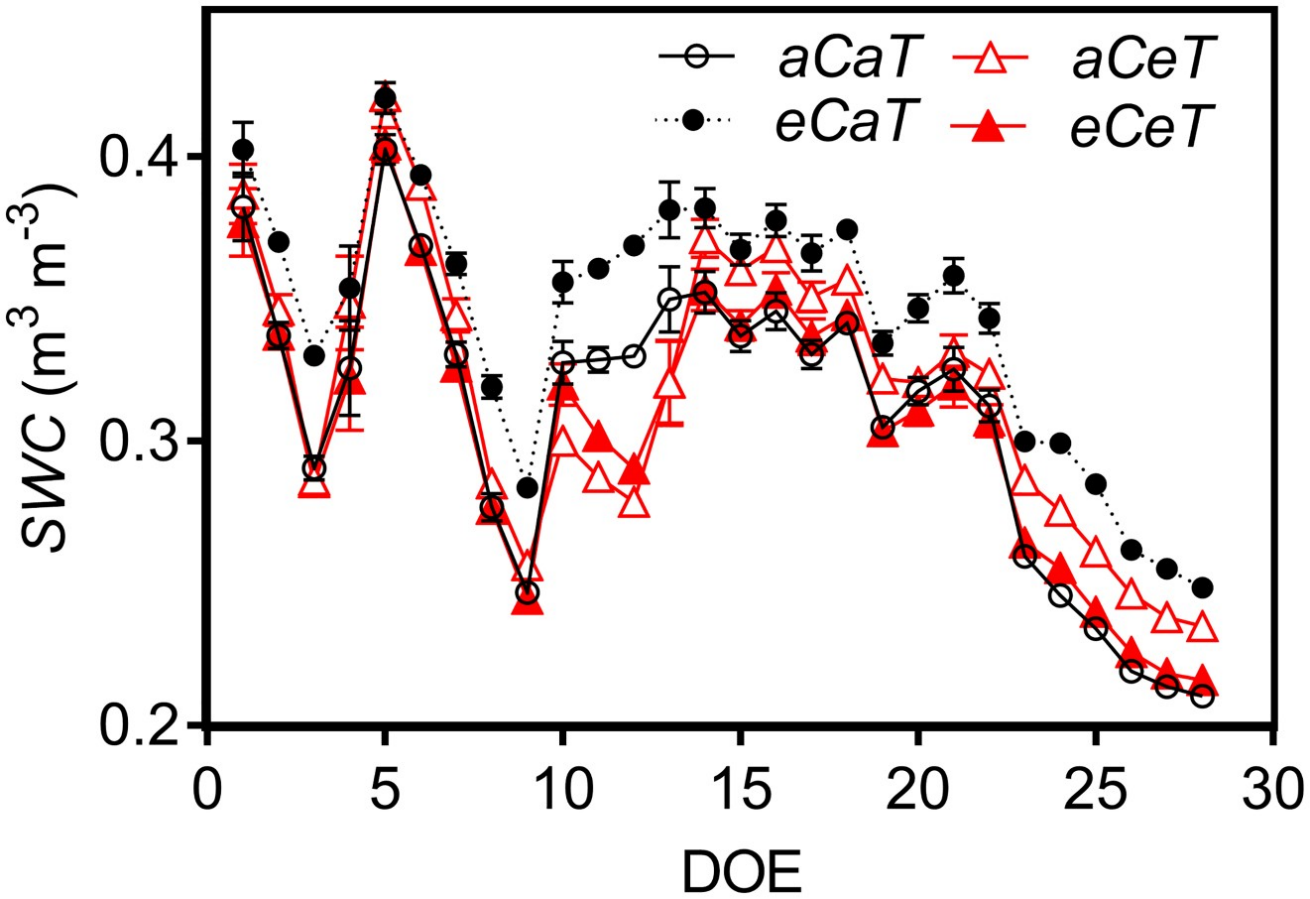
Daily average soil water content (*SWC*) registered during the experimental period at the Trop-T-FACE facility. Stack bars show the standard error. [CO_2_] levels: *aC* (ambient [CO_2_], ~400 μmol mol^-1^) and *eC* (elevated [CO_2_], ~600 μmol mol^-1^). Temperature levels: *aT* (ambient temperature) and *eT* (+2°C more than the ambient temperature).

The volumetric soil water content (*SWC*) was continually monitored during the treatment period of the experiment using Theta Probe ML2X sensors (Delta-T Devices Ltd., Burwell, Cambridge, UK) located in the center of each plot, 10 cm deep and connected to a data logger DL2e (Delta-T Devices Ltd., Burwell, Cambridge, UK). The *SWC* was conserved under *eCaT* (Fig 1). The average *SWC* was 0.30 m^3^ m^-3^ under *aCaT* and 0.34 m^3^ m^-3^ under *eCaT*. However, under *eCeT*, air warming completely canceled the conservation effect of elevated [CO_2_] on *SWC*, leading to average values of 0.31 m^3^ m^-3^ under *aCeT* and 0.30 m^3^ m^-3^ under *eCeT* (Fig 1).

### Anatomical analysis

The anatomical analysis was performed at the end of the experiment on the central region of fully expanded leaves located in the fourth node from the base to the top. Samples were collected between 08:00–12:00 h. To study the effects of experimental conditions on the ultrastructural traits, the leaf fragments were fixed in a 2% formaldehyde and 1% glutaraldehyde solution, in a 0.1 M sodium phosphate buffer (pH 7.2) [28] for 24 h. Then, samples were washed in a 0.1 M phosphate buffer solution (pH 6.8–7.0), and post-fixed in osmium tetroxide 1% for 2 h. Leaf segments were dehydrated in an acetone series and embedded in Araldite 6005. Ultrathin cross-sections (~60 nm) were obtained using an ultramicrotome (Leica Reichert) with a diamond knife (DIATOME 45) and contrasted with 2% uranyl acetate for 15 min [29] and lead citrate for 15 min [30]. Samples were observed using a Jeol JEM-100 CX-II electronic transmission microscope (JEOL, Peabody, MA, USA).

To study the effects of treatments on stomatal distribution, the leaf fragments of three leaves per plot were fixed in 50% FAA [31] for 24 h, washed in 50% ethanol for 2 h and stored in 70% ethanol until analysis. The epidermis was detached using a 1:1 solution of glacial acid acetic and hydrogen peroxide, and maintained overnight at 60°C [32]. Samples were stained with methylene blue for 10 min, mounted onto slides with 50% glycerin, and digitally photographed with a Leica DFC500 digital camera (Leica, Heidelberg, Germany) coupled with a Leica DM4000-B light microscope (Leica, Heidelberg, Germany). We evaluated five different visual fields from each leaf surface in each sample. The stomatal length (*SL*) was measured at the maximum polar length of guard cells of five stomata per visual field. We counted the number of epidermal cells and stomata and calculated the stomatal density (*SD*) and stomatal index (*SI*) (Eq 1):

$$SI(\%)=\frac{SN}{SN+EC}*100,$$ (1)

where *SN* is the stomata number, and *EC* is the number of epidermal cells.

For leaf tissue thickness measurements, we used three samples per plot fixed in 50% FAA, as described above. The leaf middle sections of each sample were kept in terpineol oil for 2 d and embedded in 2-hydroxyethyl methacrylate (Leica Instruments, Heidelberg, Germany). Cross-sections (3 μm) were cut with a Leica RM2245 microtome (Leica, Heidelberg, Germany), stained with 1% toluidine blue for 10 min [33], mounted onto slides with 50% glycerin and digitally photographed with a Leica DFC500 digital camera coupled with a DM4000-B light microscope (Leica, Heidelberg, Germany).

The measurements were always performed using ImageJ software [34] between the fourth secondary vascular bundle starting after the major vein of the leaves until the fourth secondary vascular bundle starting from the leaf margins. This region was chosen due to the greater uniformity of leaf thickness along the cross-section. From each sample, we evaluated five cross-sections and measured all the variables at three different points in each cross-section. The epidermal cells were measured at the same position in all samples using the bulliform cells as a reference. The total leaf thickness was obtained by measuring the leaf thickness of the costal and intercostal regions [35] and an average value was calculated. The mesophyll thickness was measured at the intercostal zones between the two epidermises. Moreover, we measured the distance between the vascular bundles and the polar length of the vascular bundles, vascular bundle sheath cells, bulliform cells, and sclerenchyma tissue below the vascular bundles.

### Leaf gas exchange

We performed in situ measurements of leaf gas exchange in four fully expanded leaves per plot using the LCProSD+ advanced photosynthesis measurement system (ADC BioScientific, UK) at 8 and 22 d after the treatments started, between 09:00–11:00 h. Leaves were kept in the chamber until the variables remained stable. The measurements were performed with a constant radiation of 1740 μmol m^-2^ s^-1^, temperature of 30°C (*aT* plots) or 32°C (*eT* plots), and [CO_2_] of 400 μmol mol^-1^ (*aC* plots) or 600 μmol mol^-1^ (*eC* plots). Thus, we determined the net photosynthesis rate (*A*), stomatal conductance (*g*_s_), and transpiration rate (*E*), and calculated the intrinsic water-use efficiency (*iWUE*, *A*/*g*_s_).

### Image chlorophyll fluorescence

At the end of the experiment, we measured the effective PSII quantum yield (Y[II]) using an imaging-PAM M-series chlorophyll fluorescence system MINI-version (Heinz Walz GmbH, Germany) [2, 36]. The leaves were detached, maintained in water, and dark-adapted for 20 min under room temperature. First, we determined the dark fluorescence yield (*F*_o_) (measured under a low frequency of pulse-modulated measuring light) and the maximal fluorescence yield (*F*_m_) (measured under saturation pulse). Then, the fluorescence yield (*F*) and maximal fluorescence yield in the illuminated samples (*F*_m_’) were measured. We produced images of the effective PSII quantum yield of the illuminated samples (Y[II]) with the Imaging Win software. The Y(II) was calculated as follows: Y(II) = (*F*_m_’ − *F*)/*F*_m_’.

### Malondialdehyde content

The lipid peroxidation of membranes was evaluated using the thiobarbituric acid (TBA) method of measuring the malondialdehyde (MDA) production [37]. Sampling for MDA content was performed at the end of the experiment, at the same time as anatomical samplings. First, 100 mg of fresh leaves was macerated in liquid nitrogen and solubilized in 2.5 mL of 0.1% trichloroacetic acid (TCA) (m/v). An aliquot (500 μL) of the supernatant was transferred to sealed glass tubes, in which 2 mL of 20% TCA with 0.5% TBA was added. The solution was mixed and warmed at 95°C for 30 min and put on ice to stop the reaction. The supernatant absorbance was determined at 532 and 600 nm. The reaction was performed in duplicate for each plot. The MDA concentration was calculated using a coefficient of molar extinction of 155 mM^-1^ cm^-1^ [37].

### Starch content

Starch quantification was performed on leaves collected at the end of experiment between 08:00–12:00 h, at the same time as anatomical samplings. We macerated 0.1 g of lyophilized leaves in liquid nitrogen. The material was previously extracted with 1 mL of 80% ethanol and boiled for 15 min at 80°C. This process was repeated successively three times. After centrifugation (1000 × *g*, 20 min) supernatants containing soluble sugars were discarded and residues were washed with distilled water, centrifuged, lyophilized, and used for starch quantification.

We estimated the starch content using an enzymatic analysis [38] in duplicate for each plot. We used 10 mg of lyophilized residue after ethanol extraction. Samples were incubated at 75°C for 30 min with 0.5 mL (120 U mL^-1^) of α-amylase from *Bacillus licheniformis* (EC 3.3.1.1; Megazyme, Ireland) and 10 mM 3-(N-morpholino) propanesulfonic acid (MOPS) buffer solution (pH 6.5). This process was repeated to produce a total of 120 U of enzymes. Samples were cooled to 50°C and incubated twice consecutively with 30 U mL^-1^ of amyloglucosidase from *Aspergillus niger* (EC 3.2.1.3; Megazyme, Ireland) in 500 μL of 0.1 M sodium acetate buffer (pH 4.5) for 30 min. The reaction was stopped by the addition of 100 μL of 0.8 M perchloric acid. After centrifugation (10,000 x *g*, 2 min), aliquots of the supernatant were incubated with the reagent Glucose PAP Liquiform (Centerlab) containing glucose oxidase and peroxidase (GOD-POD), 4-aminoantipyrine 50 mM and phenol pH 7.5. After incubation for 14 min at 37°C, the glucose content was determined in an ELISA plate at 490 nm.

### Dry mass

At the end of the experiment, intact plants of a 50-cm diameter circular sampling area were harvest at ground level. We separated leaves and stems and dry the material in an oven at 60°C until constant weight. We calculated leaf, stem and total aboveground biomass (g m-^2^).

### Data analysis

For quantitative data, we used a 2 × 2 factorial analysis of variance (ANOVA) (two factors with two levels) to test the main effects of [CO_2_] and temperature, as well as their interaction when factors were combined. Means of significant interactive effects were compared using a post-hoc Student’s *t*-test. In order to meet the ANOVA assumptions, the data were log-transformed when necessary. Analyses were performed using R software 3.2.3 [39], with a significance level of 5% (*p* < 0.05).

## Results

### Ultra-structural analysis

**Fig 2:**
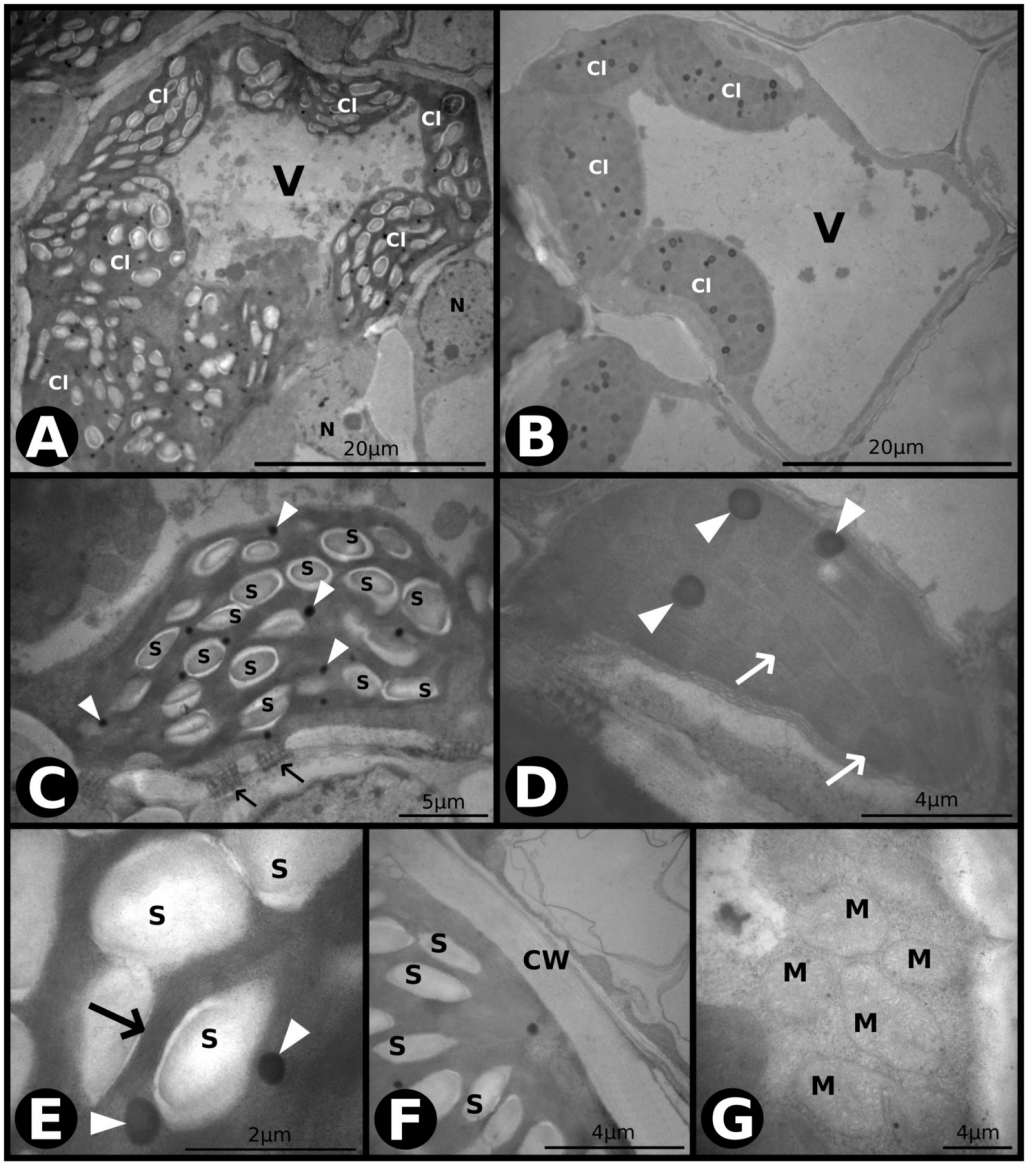
Transmission electron microscopy (TEM) photomicrographs of leaves of *P*. *maximum* under ambient [CO_2_] and ambient temperature (*aCaT*). (A) Overview of a bundle sheath cell (BSC). Cl–chloroplast, V–vacuole. N–nucleus. (B) Overview of a mesophyll cell (MC). Cl–chloroplast, V–vacuole. (C) A chloroplast of a BSC with plastoglobuli (indicated by arrowheads) and starch grains. Arrows indicate plasmodesmata between the BSC and MC. S–starch. (D) A chloroplast of a MC with plastoglobuli (indicated by arrowheads) and thylakoid membranes (indicated by arrows). (E) A chloroplast of a BSC showing details of thylakoid membranes (indicated by arrow) and plastoglobuli (indicated by arrowheads). S–starch. (F) The cell wall between the BSC and MC. S–starch, CW–cell wall. (G) Group of mitochondria of a BSC. M–mitochondria.

Qualitative ultra-structural analysis showed that only the bundle sheath cells (BSC) were affected by the experimental conditions. Under *aCaT*, the chloroplast thylakoid membranes in the BSC were well developed, with numerous plastoglobuli and starch grains (Fig 2A, 2C, 2E and 2F). The mitochondria (Fig 2G) and cell wall were regular (Fig 2C and 2F) with many cell-to-cell communications through the plasmodesmata occurring between the BSC and MC (Fig 2C). At the mesophyll cells (MC), no starch accumulation was observed, and thylakoid membranes were well developed, with many plastoglobuli and a multilayer external membrane of chloroplasts (Fig 2B and 2D).

**Fig 3:**
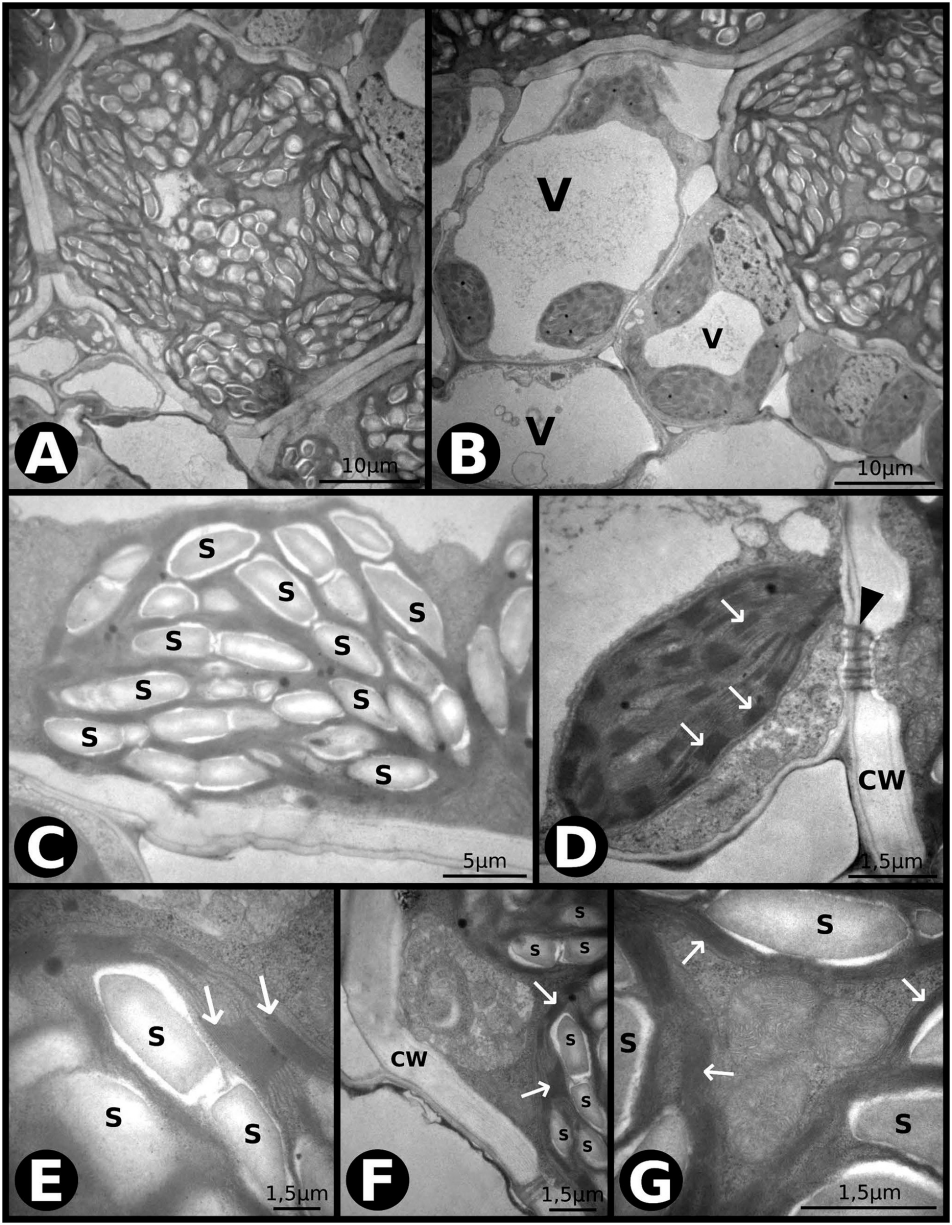
Transmission electron microscopy (TEM) photomicrographs of leaves of *P*. *maximum* under elevated [CO_2_] and ambient temperature (*eCaT*). (A) Overview of a bundle sheath cell (BSC). (B) Overview of mesophyll cells (MC). V–vacuole. (C) Chloroplast of a BSC with large starch grains. S–starch. (D) Chloroplast of a MC with conserved thylakoid membranes (indicated by arrows) and communication thought plasmodesmata with a BSC (indicated by an arrowhead). CW–cell wall. (E) A chloroplast of a BSC showing details of thylakoid membranes (indicated by arrow). S–starch. (F) Regular cell wall of a BSC and chloroplasts with starch grains and conserved thylakoid membranes (indicated by arrows). CW–cell wall, S–starch. (G) Group of mitochondria associated with chloroplasts in BSC with conserved thylakoid membranes (indicate by arrows). S–starch, M–mitochondria.

Under *eCaT*, the chloroplasts of the BSC were more prominent with large starch grains (Fig 3), filling in most of the chloroplast cross-section area (Fig 3A and 3C). The thylakoid membranes of the BSC were intact under this treatment (Fig 3E, 3F and 3G), and chloroplasts and mitochondria were frequently observed in association (Fig 3G). The mitochondria (Fig 3G) and cell wall were regular (Fig 3F), with many cell-to-cell communications occurring between the two cell types (Fig 3D). We found less dense and numerous plastoglobuli in chloroplasts of the BSC (Fig 3A, 3C and 3E) compared to those under *aCaT* (Fig 3A, 3C and 3E). In the MC, no alterations were observed, and all structures were regular (Fig 3B and 3D).

**Fig 4:**
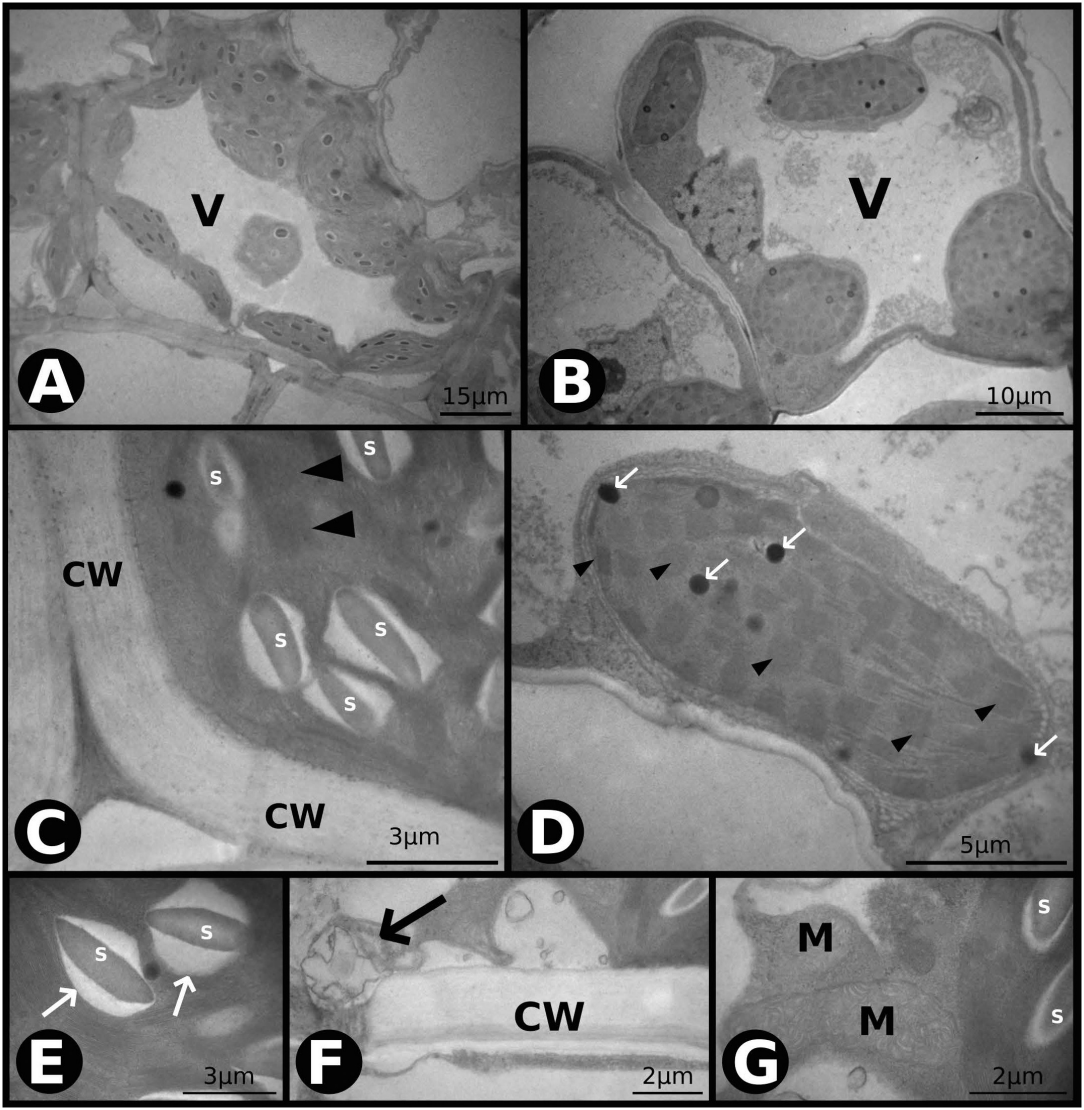
Transmission electron microscopy (TEM) photomicrographs of leaves of *P*. *maximum* under ambient [CO_2_] and air warming (*aCeT*). (A) Overviews of a bundle sheath cell (BSC). V–vacuole. (B) Overview of a mesophyll cell (MC). V–vacuole. (C) Chloroplast of a BSC with spaces between the starch grains and chloroplasts and conserved thylakoid membranes (indicated by arrowheads). CW–cell wall, S–starch. (D) Overview of a chloroplast of a MC with plastoglobuli (indicated by arrows) and conserved thylakoid membranes (indicated by arrowheads). (E) Thylakoid membrane detail of the chloroplast of a BSC and spaces between the starch grains and chloroplasts (indicated by arrows). S–starch. (F) Cytoplasm retraction in a BSC and traffic of vacuoles with an MC through plasmodesmata (indicated by an arrow). CW–cell wall. (G) Association of mitochondria and chloroplasts of a BSC. S–starch, M–mitochondria.

Under *aCeT*, we found minor and less bulky starch grains in the chloroplasts of BSC (Fig 4A, 4C and 4E), when compared to *aCaT* (Fig 2A, 2C and 2E) and *eCaT* (Fig 3A, 3C and 3E). Chloroplasts of BSC were smaller (Fig 4A) with conserved thylakoid membranes (Fig 4C and 4E). Chloroplasts and mitochondria were frequently observed in association with BSC (Fig 4G), and space was observed between the chloroplasts and starch grains (Fig 4C and 4E). In the BSC, we observed cytoplasm retraction and traffic of vacuoles with MC (Fig 4F). The MC showed conserved thylakoid membrane structures, many plastoglobuli, and a multilayer external membrane of chloroplasts (Fig 4B and 4D).

**Fig 5:**
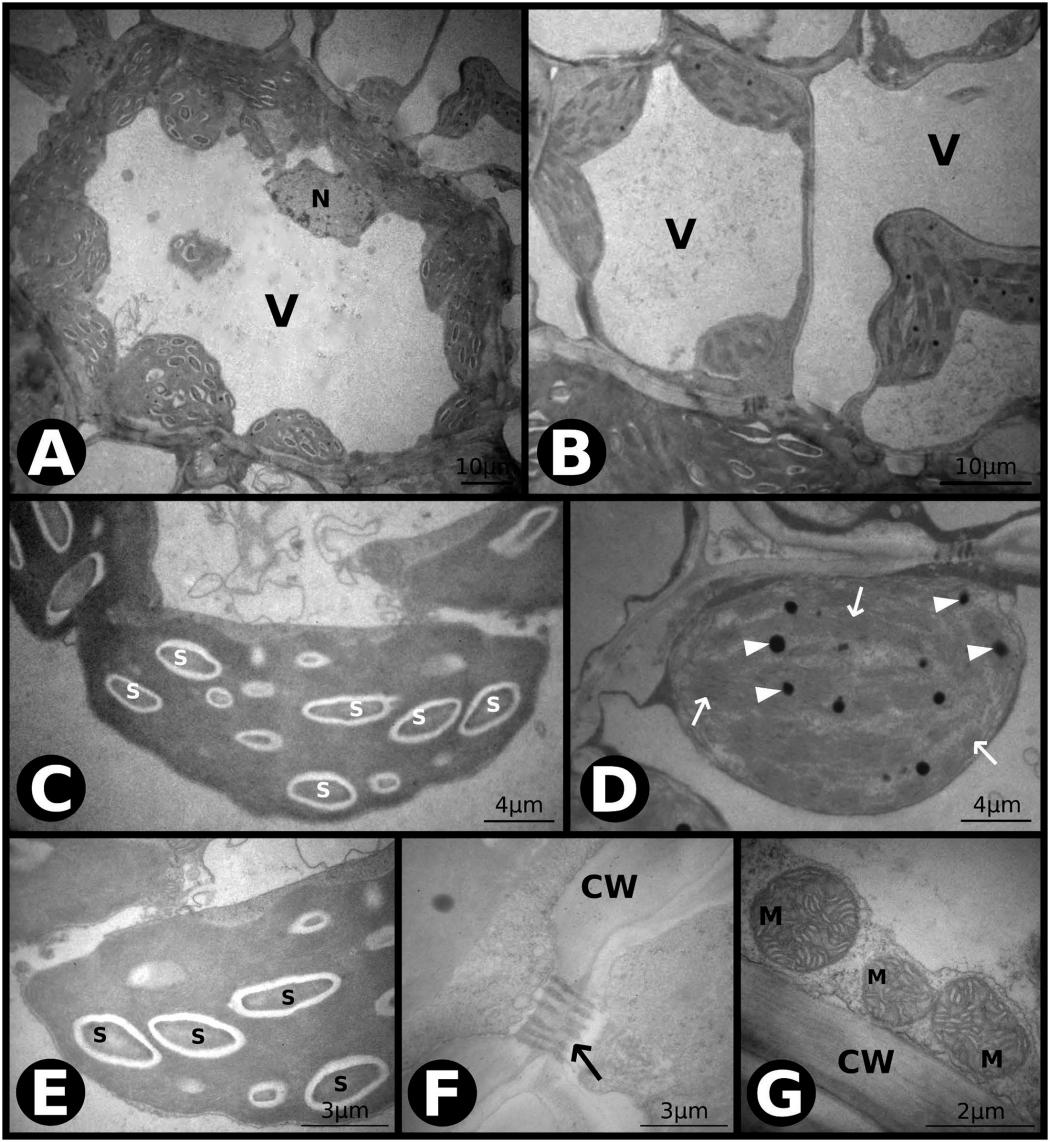
Transmission electron microscopy (TEM) photomicrographs of leaves of *P*. *maximum* under elevated [CO_2_] and air warming (*eCeT*). (A) Overview of a bundle sheath cell (BSC). V–vacuole, N–nucleus. (B) Overview of mesophyll cells (MC). V–vacuole. (C) Chloroplast of a BSC with starch grains. S–starch. (D) Chloroplast of a MC with plastoglobuli (indicated by arrowheads) and conserved thylakoid membranes (indicated by arrows). (E) Details of the thylakoid membrane and external membrane of chloroplast of a BSC. S–starch. (F) Plasmodesmata (indicated by an arrow) between a BSC and a MC with regular cell wall. CW–cell wall. (G) Group of mitochondria with conspicuous cristae. M–mitochondria, CW–cell wall.

Under *eCeT*, BSC showed a reduced size and number of chloroplasts, with loss of integrity in the external membrane of chloroplasts (Fig 5A, 5C and 5E) but with a conserved structure of thylakoids (Fig 5C and 5E). On these chloroplasts, starch grains are small and less numerous (Fig 5A, 5C and 5E) when compared to those under *aCaT* (Fig 2A, 2C and 2E) and *eCaT* (Fig 3A, 3C and 3E). Associations between mitochondria and chloroplasts were not observed in this treatment. However, mitochondria cristae were more conspicuous (Fig 5G). In MC, no alterations were observed, and all structures were regular (Fig 4B and 4D).

### Thickness of tissues and cells

Leaf cross-sections were used to study the effects of experimental conditions on the thickness of the leaf tissues and cells (Table 1). Air warming exerted more pronounced effects on the thickness of tissues than did elevated [CO_2_]. Leaves grown under *eT* independent of [CO_2_] level showed an adaxial cuticle (*CU*_*AD*_) that was approximately 13% thicker, while our experimental conditions did not change the abaxial cuticle (*CU*_*AB*_). The adaxial epidermis (*EP*_*AD*_) thickness was increased by 11% under *eC*, regardless of the temperature level, with no alterations at the abaxial surface (*EP*_*AB*_) (Table 1). Air warming reduced the total leaf thickness (*LT*) by approximately 17%, regardless of [CO_2_] level. The main effect of air warming was also observed for the polar length of the bulliform cells (*BUL*) and polar length of vascular bundles (*VAS*_*BU*_), with decreases of 11% and 15%, respectively. However, the sclerenchyma thickness (*SLC*), mesophyll thickness (*MES*), polar length of vascular bundle sheath cells (*BSC*), and distance between the vascular bundles (*VAS*_*DIS*_) did not change (Table 1).

**Table 1:** Quantitative parameters (μm) measured in leaves of *P*. *maximum* grown under different levels of [CO_2_] and temperature at Trop-T-FACE facility. *CU*_*AD*_ = adaxial cuticle thickness. *CU*_*AB*_ = abaxial cuticle thickness. *EP*_*AD*_ = adaxial epidermis thickness. *EP*_*AB*_ = abaxial epidermis thickness. *SCL* = sclerenchyma thickness. *MES* = mesophyll thickness. *LT* = leaf thickness. *BUL* = polar length of bulliform cells. *VAS*_*BU*_ = polar length of vascular bundles. *BSC* = polar length of bundle sheath cells. *VAS*_*DIS*_ = distance between vascular bundles. Param. = parameter. ANOVA = *p*-values for significant effects: [CO_2_] (isolated effect of elevated [CO_2_]), T. (isolated effect of air warming), and [CO_2_] × T. (interaction of elevated [CO_2_] × Temp). [CO_2_] levels: *aC* (ambient [CO_2_], ~400 μmol mol^-1^) and *eC* (elevated [CO_2_], ~600 μmol mol^-1^). Temperature levels: *aT* (ambient temperature) and *eT* (2°C more than the ambient temperature).

	Atmospheric [CO_2_] level				ANOVA		
	*aC*		*eC*				
Param.	Temperature level		Temperature level		[CO_2_]	T.	[CO_2_]×T
	*aT*	*eT*	*aT*	*eT*			
*CU*_*AD*_	2.98 ± 0.14	3.32 ± 0.05	2.87 ± 0.10	3.10 ± 0.08	ns	*	ns
*CU*_*AB*_	2.80 ± 0.02	2.86 ± 0.11	2.93 ± 0.02	2.86 ± 0.04	ns	ns	ns
*EP*_*AD*_	16.42 ± 0.62	16.42 ± 0.10	18.59 ± 0.53	17.35 ± 0.11	*	ns	ns
*EP*_*AB*_	17.36 ± 0.21	17.18 ± 0.46	16.7 ± 0.39	17.76 ± 0.59	ns	ns	ns
*SCL*	17.82 ± 0.41	17.20 ± 0.54	18.23 ± 0.42	17.94 ± 0.73	ns	ns	ns
*MES*	88.06 ± 1.85	77.28 ± 2.60	79.31 ± 4.69	81.07 ± 3.86	ns	ns	*
*LT*	207.75 ± 1.74	162.19 ± 10.06	194.18 ±14.50	184.65 ± 9.53	ns	*	*
*BUL*	55.27 ± 0.70	46.80 ± 1.50	51.81 ± 2.95	49.28 ± 2.07	ns	ns	*
*VAS*_*BU*_	48.26 ± 1.39	40.10 ± 0.95	45.47 ± 3.27	42.27 ± 1.60	*	*	**
*BSC*	64.09 ± 1.01	59.89 ± 1.32	62.92 ± 2.68	64.42 ± 3.23	ns	ns	ns
*VAS*_*DIS*_	176.52 ± 4.82	174.65 ± 1.92	179.76 ± 6.06	180.75 ± 3.69	ns	ns	ns

Data are mean ± SD (n = 4).

ns = not significant.

*, *p* < 0.05;

**, *p* < 0.001;

***, *p* < 0.001.

### Stomatal parameters

**Fig 6:**
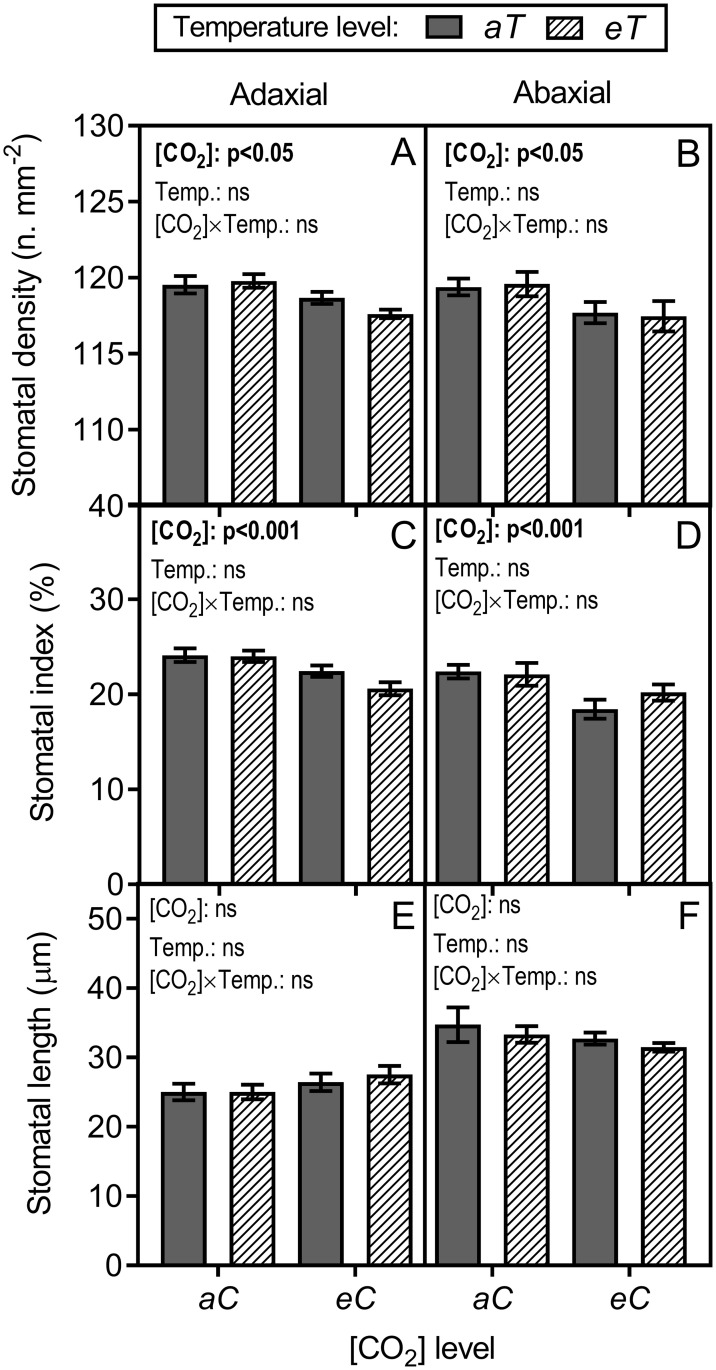
Stomatal parameters measured in leaves of *P*. *maximum*. (A) Adaxial and (B) abaxial stomatal density–*SD*. (C) Adaxial and (D) abaxial stomatal index–*SI*. (E) Adaxial and (F) abaxial stomatal length–*SL*. Measurements are shown for both leaf surfaces, and statistical analysis was performed between treatments on same leaf surface. Stack bars show the standard error. [CO_2_] levels: *aC* (ambient [CO_2_], ~400 μmol mol^-1^) and *eC* (elevated [CO_2_], ~600 μmol mol^-1^). Temperature levels: *aT* (ambient temperature) and *eT* (2°C more than the ambient temperature). ANOVA *p*-values are shown and significant effects (*p* < 0.05) are detailed in bold. [CO_2_] (isolated effect of elevated [CO_2_]), Temp. (isolated effect of air warming) and [CO_2_] × Temp. (interaction of elevated [CO_2_] × Temp.).

We observed that a CO_2_-enriched atmosphere significantly affected the stomata differentiation on both leaf surfaces (Fig 6). Thereby, the adaxial stomatal density (*SD*) and stomatal index (*SI*) decreased under *eC* by approximately 6% for both variables, independent of temperature level (Fig 6A and 6C). At the abaxial leaf surface, the *SD* and *SI* decreased under *eC*, regardless of temperature level, by 16% and 11%, respectively (Fig 6B and 6D). Air warming had no effects on the stomatal distribution (Fig 6A, 6B, 6C and 6C). The stomatal size (*SL*) was not affected by the experimental conditions (Fig 6E and 6F), and no interactions between elevated [CO_2_] × air warming were observed for any of the stomatal parameters (Fig 6).

### Leaf gas exchange

**Fig 7:**
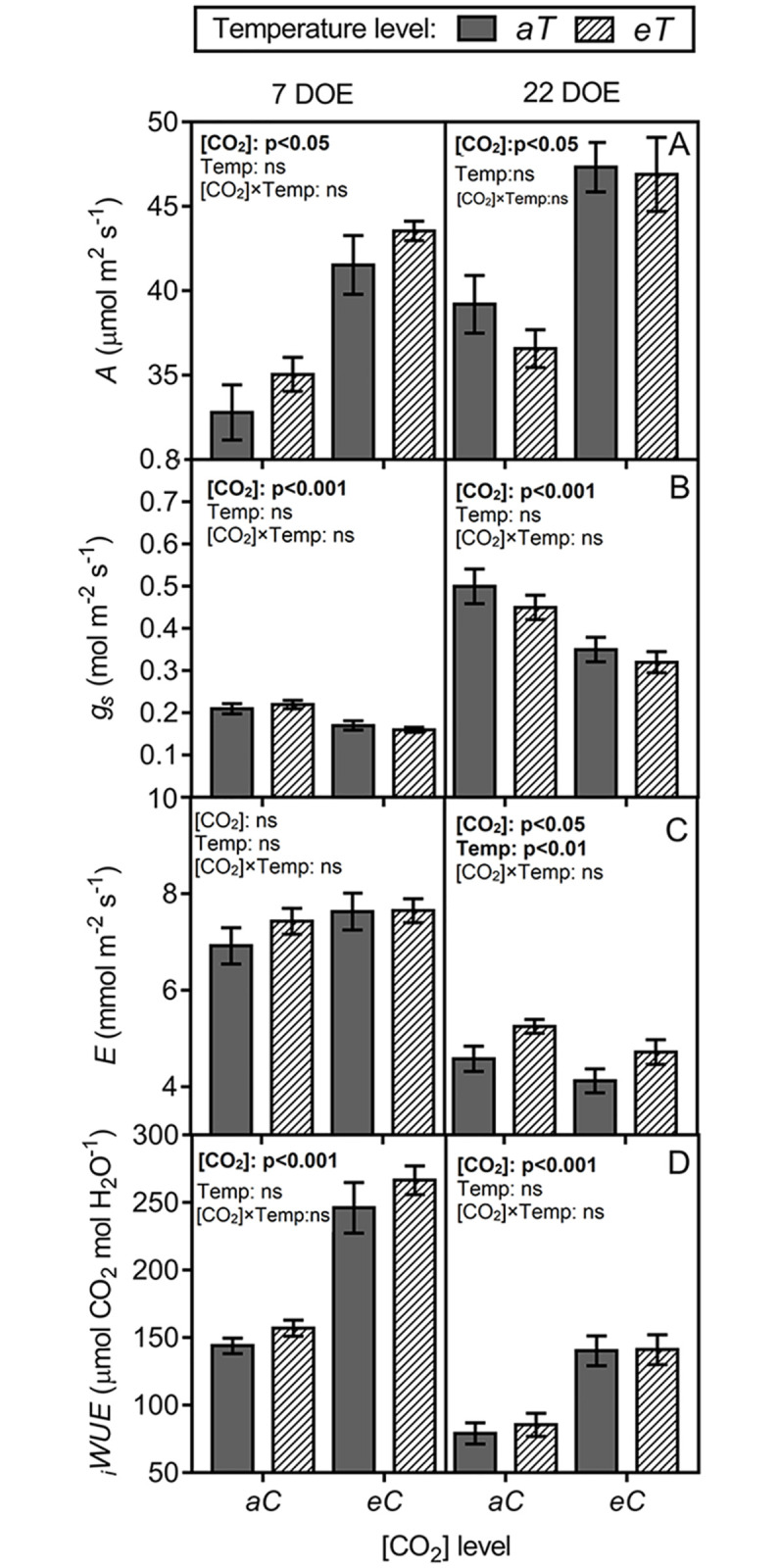
Leaf gas exchange parameters measured during the growing season of *P*. *maximum* at the Trop-T-FACE facility. (A) Net photosynthesis rate (*A*). (B) Stomatal conductance (*g*_*s*_). (C) Transpiration rate (*E*). (D) Intrinsic water-use efficiency (*iWUE*). Stack bars show the standard error. [CO_2_] levels: *aC* (ambient [CO_2_], ~400 μmol mol^-1^) and *eC* (elevated [CO_2_], ~600 μmol mol^-1^). Temperature levels: *aT* (ambient temperature) and *eT* (2°C more than the ambient temperature). ANOVA *p*-values are shown and significant effects (*p* < 0.05) are detailed in bold. [CO_2_] (isolated effect of elevated [CO_2_]), Temp. (isolated effect of air warming), and [CO_2_] × Temp. (interaction of elevated [CO_2_] × Temp.).

We measured the leaf gas exchange for 2 d of the 30-d experiment (Fig 7). Increased levels of atmospheric [CO_2_] exerted more pronounced effects on the gas exchange parameters than did air warming. The net photosynthesis rate (*A*) increased under *eC* independent of temperature level by approximately 25% on both sampling days (Fig 7A). Along with stomatal density reductions under elevated [CO_2_], the stomatal conductance (*g*_*s*_) was also decreased under *eC* regardless of temperature level by approximately 23% and 30% at 7 and 22 d of experiment (DOE), respectively (Fig 7B). Air warming had a marginal effect on leaf transpiration rate (*E*). At 7 DOE, no effects were observed. However, at 22 DOE, we detected an antagonistic effect between the factors. Under *eCaT*, *E* was decreased by 10%, whilst under *aCeT*, *E* was increased by 15%; therefore, the mean *E* values between *aCaT* and *eCeT* did not differ (Fig 7C). The combination of increased leaf-level carbon assimilation and reduced *g*_*s*_ under *eC*, increased water use efficiency (_*i*_*WUE*) regardless of the temperature level by 70% and 71% at 7 and 22 DOE, respectively (Fig 7D).

### Chlorophyll fluorescence and malondialdehyde content

At the end of the experiment, we measured the effective Y(II) (S2 Fig), which presented values > 0.72, and the malondialdehyde content (MDA) (S3 Fig). We observed that both parameters were not affected by the air warming and elevated [CO_2_] treatments.

### Starch content

**Fig 8:**
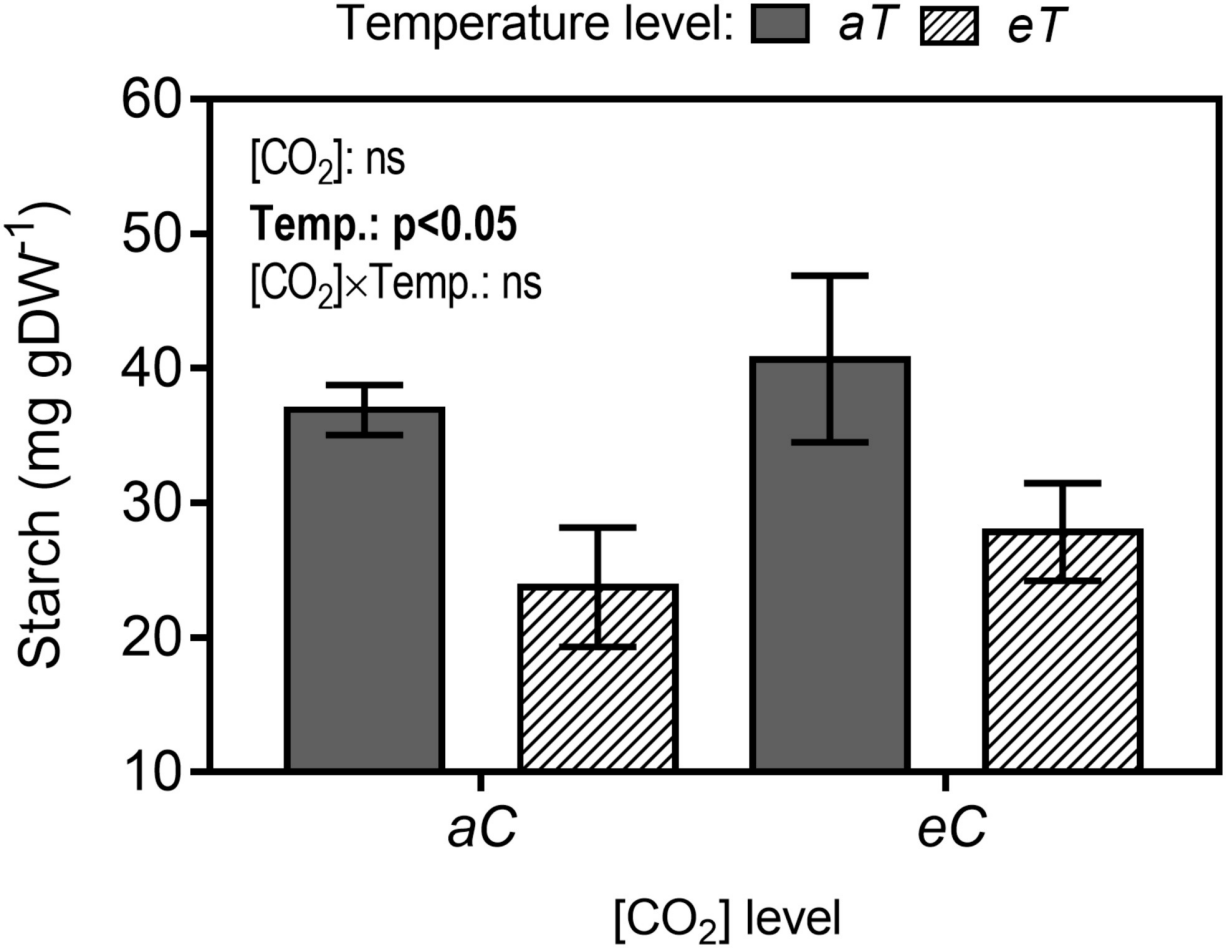
Starch content in leaves of *P*. *maximum* at Trop-T-FACE facility. Stack bars show the standard error. [CO_2_] levels: *aC* (ambient [CO_2_], ~400 μmol mol^-1^) and *eC* (elevated [CO_2_], ~600 μmol mol^-1^). Temperature levels: *aT* (ambient temperature) and *eT* (2°C more than the ambient temperature). ANOVA p-values are show and significant effects (*p* <0.05) are detailed in bold. [CO_2_] (isolated effect of elevated [CO_2_]), Temp. (isolated effect of air warming) and [CO_2_] × Temp. (interaction of elevated [CO_2_] × Temp.).

Leaf starch content was estimated in samples collected at the same time as leaf anatomical samplings. Enzymatic assay confirmed our qualitative results of Figs 4A, 4C, 4E, 5A, 5C and 5E. Thus, under *eT* and independent of [CO_2_] level, starch content was reduced in approximately 30% (Fig 8).

### Dry mass

**Fig 9:**
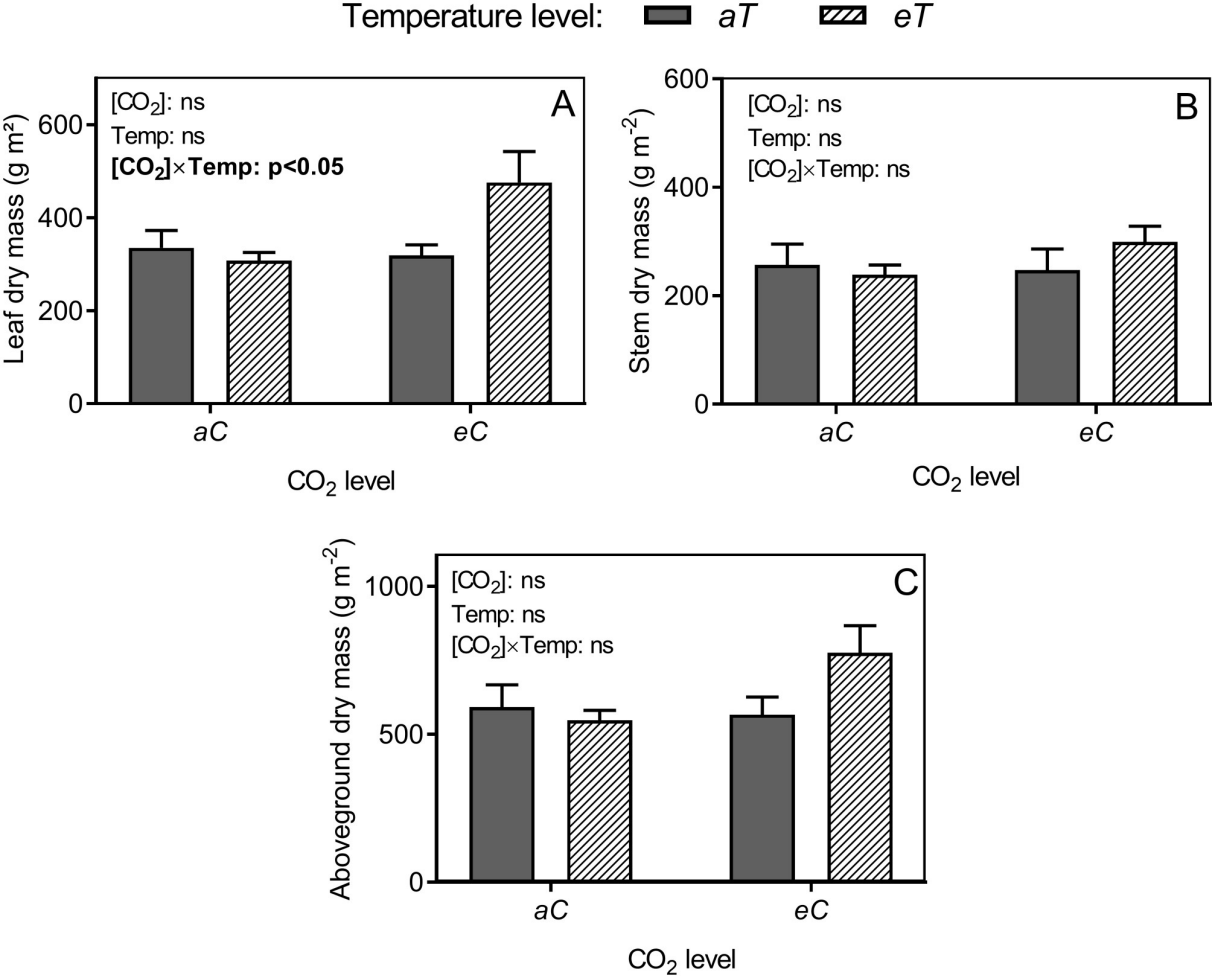
Dry mass of *P*. *maximum* at Trop-T-FACE facility. (A) Leaf dry mass. (B) Stem dry mass. (C) Total aboveground biomass. Stack bars show the standard error. [CO_2_] levels: *aC* (ambient [CO_2_], ~400 μmol mol^-1^) and *eC* (elevated [CO_2_], ~600 μmol mol^-1^). Temperature levels: *aT* (ambient temperature) and *eT* (2°C more than the ambient temperature). ANOVA p-values are show and significant effects (*p* <0.05) are detailed in bold. [CO_2_] (isolated effect of elevated [CO_2_]), Temp. (isolated effect of air warming) and [CO_2_] × Temp. (interaction of elevated [CO_2_] × Temp.).

Leaf dry mass was enhanced in approximately 42% under *eCeT* (interactive effect) (Fig 9A). However, no effects were observed for stem and total aboveground biomass (Fig 9B and 9C).

## Discussion

**Fig 10:**
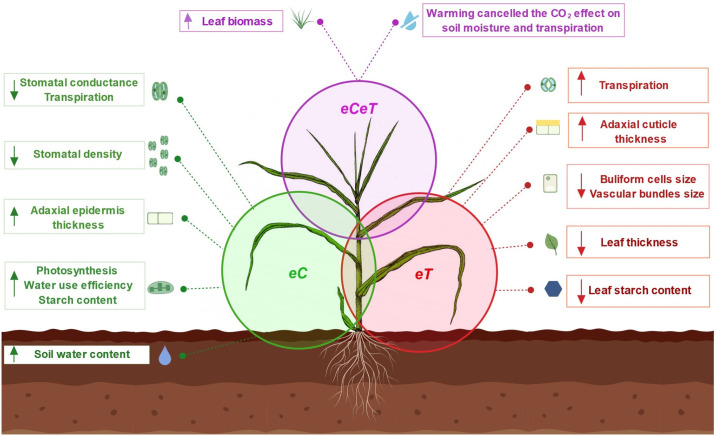
Main anatomical and physiological acclimation mechanisms of *P*. *maximum* developed under elevated [CO_2_] and warming. Created with BioRender. Elevated [CO_2_] (*eC*, green circle, isolated effect of CO_2_) exerted more pronounced effects on epidermis anatomy and leaf gas exchange. A CO_2_-enriched atmosphere reduced the differentiation of epidermal cells to stomata on both leaf surfaces, reducing stomatal density and index. In addition, stomatal aperture and transpiration were also decreased. Therefore, water use efficiency, photosynthesis and starch content increased. Due to low transpiration flux, soil water content was conserved during the experiment. Warming (*eT*, red circle, isolated effect of temperature) affected leaf structure and starch metabolism. Leaves developed protection mechanisms against the effects of a warmer environment with a thicker adaxial cuticle and reduced size of vascular bundles and bulliform cells. Under the combination of elevated [CO_2_] and warming (*eCeT*, purple circle, interaction of CO_2_ × temperature), warming cancelled the CO_2_ effect on soil water content and transpiration. However, when combined, these two environmental factors produced a set of anatomical adjustments that contributed to the acclimation of this species to future conditions increasing leaf biomass production. Down arrow: decrease. Up arrow: increase.

In earlier experiments carried out by our team [3], it was found that elevated temperature (+2°C above ambient) with adequate water availability, increased leaf area and leaf biomass production of *P*. *maximum*, while the elevated atmospheric CO_2_ concentration ([CO_2_]) (~600 ppm) reduced the leaf/stem ratio biomass of this species. In this work, we reported the effects of warming and elevated [CO_2_] on the ultrastructure, leaf anatomy, and physiology of *P*. *maximum* growing at the Trop-T-FACE facility. Here, we identified the main anatomical mechanisms of acclimation in *P*. *maximum* leaves developed under elevated [CO_2_] and air warming under field conditions (Fig 10). We found that a CO_2_-enriched atmosphere significantly reduced the differentiation of epidermal cells to stomata on both leaf surfaces. The parallel changes in *SD* and *SI* exclude the possibility of changes in the epidermal cell size or number (Fig 6), as observed in maize plants (*Zea mays*, C4) grown under 700 μmol mol^-1^ of [CO_2_] [14]. However, in *Panicum antidotale* (Poaceae, C4) and *Panicum decipiens* (Poaceae, intermediate C3/C4), stomatal density increased under 900 μmol mol^-1^ of [CO_2_], whilst in *Panicum tricanthum* (C3) *SD* decreased, suggesting different acclimation mechanisms under elevated [CO_2_] between *Panicum* species [13]. Stomatal number control is a protective mechanism that enhances the utilization of water resources under elevated [CO_2_] [40, 41, 42]. This response is considered a long-term acclimation mechanism that occurs only when leaves are fully developed under a CO_2_-enriched atmosphere. The influence of stomatal density on leaf gas exchange was studied in detail in stomatal mutants of *Arabidopsis thaliana* (Brassicaceae) [43], where fewer stomata on the leaf surfaces were responsible for decrease the stomatal conductance (*g*_s_) and transpiration rate (*E*).

The gas exchange also changed as a result of short-term mechanisms, such as stomatal closure, which is considered one of the most important factors that reduce *g*_s_ under elevated [CO_2_] [44]. In this study, *g*_s_ decreased under *eC* regardless of temperature level, probably due to a combination of fewer stomata and reduced pore aperture induced by elevated [CO_2_]. As a consequence, we observed lower transpiration rates under this treatment (Fig 7); this effect was corroborated by our *SWC* data (Fig 1). The lower transpiration rates by plants under *eCaT* conserved soil moisture because soil water uptake by the roots was also reduced [45, 46], since transpiration flux was low. It is accepted that soil moisture conservation can benefit plants, especially during water shortages periods [47–49]. However, under *eCeT*, air warming counterbalanced the effects of *eC* on *E* and *SWC*, presumably due to the increased evapotranspiration.

Although we observed that the increased size of epidermal cells did not cause a reduced stomatal density, the cross-sectional thickness of the adaxial epidermis increased under *eC* independent of temperature. This same pattern of response was found in leaves of *Triticum aestivum* (Poaceae), suggesting that elevated [CO_2_] affected cell growth anisotropy [50]. Cell expansion occurs due to the stimulation of elevated [CO_2_] on the enzymatic activity of xyloglucan endo-transglycosylase, which is responsible for the expansion of cell walls [51]. It has been suggested that a thicker epidermis is a xeromorphic trait, and leaves grown under a CO_2_-enriched atmosphere would be more resistant to drought since they would avoid increased transpiration in a warmer environment [52].

We found that under elevated atmospheric [CO_2_], leaf-level carbon assimilation significantly increased regardless of temperature (Fig 7A). An enhanced photosynthesis under elevated [CO_2_] is often higher in C3 than in C4 species [10, 53]. However, some C4 species are non-saturated under current [CO_2_] conditions [54]. This response was also observed in Brazilian cultures of sugarcane (*Saccharum officinarum*, C4), in which photosynthesis was increased by approximately 30% under elevated [CO_2_] [55, 56]. The enhancement in the photosynthetic performance of *P*. *maximum* resulted in more numerous and larger starch grains in chloroplasts of bundle sheath cells (Fig 3), and a non-significant increase (10%) in the leaf starch content (Fig 8). Interestingly, *Panicum antidotale* (C4) plants grown under 900 μmol mol^-1^ of [CO_2_], accumulated starch grains not only in the chloroplasts of bundle sheath cells, but also on mesophyll [13]. The conservation of chloroplast ultrastructure under elevated [CO_2_] is in agreement with previous studies performed on other *Panicum* species, such as *P*. *antidotale* (C4), *P*. *decipiens* (C3-C4), and *P*. *tricanthum* (C3) [13]. However, it has been reported that chloroplast ultrastructure can be damaged due to an excessive accumulation of starch grains [57]. This phenomenon is considered to be one of the causes of photosynthesis acclimation observed in some species exposed to elevated [CO_2_] levels [57, 58]. In this study, the gas exchange adjustments resulted in an enhanced intrinsic water-use efficiency (*iWUE*) regardless of temperature level in *P*. *maximum*. The *iWUE* is one of the most sensitive parameters that respond to elevated [CO_2_] levels, with increases of up to 229% in some species [45]. The increase in *iWUE* is presumably an optimization of the ratio unit of assimilated carbon per unit of water used under global change conditions, resulting in an amelioration of the adverse effects of drought, on plant growth [59]. Plants often achieve improved *iWUE* by producing leaves with fewer stomata [42], as observed in this study.

The ultrastructure of thylakoid membranes remained intact under warmed plots; however, we observed a loss of integrity in the external membranes of chloroplasts under *eCeT*. Although light-harvesting complexes are located in the thylakoid membranes, the external membrane may act in the transport of molecules between the cytoplasm and stroma [60]. In rice (*Oryza sativa*, Poaceae), ultrastructural damages that resulted in differences in the chlorophyll fluorescence yield were observed only under severe swelling of the thylakoid membranes, while the external membrane remained intact [61]. To investigate the possible effect of this morphological alteration on the photosynthetic process, we analyzed the chlorophyll fluorescence and MDA data measured at the same time as anatomical samplings. As observed in S2 Fig, the Y(II) was not affected in any treatment, indicating no effects of treatments on the maximum photochemical yield of photosystem II. Moreover, malondialdehyde (MDA), which is a highly reactive compound and a natural marker of oxidative stress [37] was also not affected by *eCeT* (S3 Fig). Furthermore, *A* was enhanced under *eC*, independent of temperature (Fig 7A). This set of physiological data indicated that the loss of integrity of the external membrane of chloroplasts under *eCeT* was not expressed through any measurable deleterious effect on photosynthesis.

Contradicting our main hypothesis, air warming showed no effects on photosynthesis, and carbon fixation was enhanced only by *eC*. However, we observed that starch grains in chloroplasts of BSC were smaller and less numerous under *eT* (Figs 4A, 4C, 4E, 5A, 5C and 5E), which was confirmed by starch quantification (Fig 8). Starch is the main carbohydrate stored in higher plants [62] and its content changes according to the starch–sucrose remobilization of source–sink relationships. Besides the storage function, starch is reported to be an important molecule acting in signaling networks between organs [63]. During air warming, plants remobilize starch to other part plants to provide energy to sink tissues [63]. In this study, we hypothesized that air warming acts as a stimulus to starch exportation. Reduced starch content was also observed under *eCeT*, where *A* was higher, indicating that the stimuli of starch breakdown are independent of [CO_2_] level, and surplus carbon provided by enhanced photosynthesis is also being exported, presumably enhancing dry leaf mass under *eCeT* (Fig 9A).

We observed that leaves grown under moderate air warming developed adjustments to better deal with the warmer environment. Our data showed that warmed leaves had a thicker adaxial cuticle regardless of the [CO_2_] level, presumably as a response related to an acclimation mechanism to avoid excessive water loss [64, 65]. The cuticle is a cutin hydrophobic layer, and plays an essential role in isolating plant surfaces and reducing nanoscale water diffusion [66]. Interestingly, in *Panicum antidotale* (C4) plants, elevated [CO_2_] is responsible for increased adaxial cuticle deposition [13]. Furthermore, we observed that the size of bulliform cells, which store water, was decreased under *eT*. The bulliform cells are located at the adaxial leaf surface between the vascular bundles, and are essential in the process of leaf wilting and opening by controlling its turgor [67]. By reducing the volume of these cells, plants cause leaf wilting and reduce the leaf surface exposed to sunlight, indicating a better control of water loss under warmer ambient temperatures. Excessive transpiration may lead to the cavitation of xylem conduits, causing a disruption in the water flow and threatening plant survival [68]. In this study, warmed plants showed a reduced of vascular bundle size. Smaller vascular bundles decrease the probability of xylem cavitation [69], an essential adaptation in species grown under warm environments. Besides, our data showed that *eT* lowered the leaf thickness. A 10% reduction in leaf thickness was also observed in warmed (+2°C) maize plants [15]. Thinner leaves have an increased thermal conductivity, which provides a more efficient heat dissipation with reduced water loss by evapotranspiration in warmer environments [5, 70]. Thin leaves are adaptations often found in xeromorphic species [71], since small cells support a higher negative turgor pressure than larger ones due to the relationship between the cell wall elasticity and cell volume [71, 72].

We found no evidence of interactive effects between elevated [CO_2_] and air warming in terms of anatomical responses of *P*. *maximum*. This result is consistent with those of other studies [5, 73], which suggest that leaf anatomy may include a response of simple additive effects under elevated [CO_2_] and temperature. However, under *eCeT* an interaction between CO_2_ and air warming greatly enhanced leaf biomass production. This result was presumably associated with the combination of anatomical acclimation mechanisms independently developed by elevated CO_2_ and warming. In addition, increased photosynthesis and _*i*_*WUE* performed by *eC* and starch remobilization performed by *eT* may be contributed to the production of more leaves, increasing dry leaf mass.

## Conclusions

To our knowledge, this study was the first to provide evidence of the physiological and anatomical mechanisms of acclimation in *P*. *maximum* leaves growing in a tropical environmental under future conditions of elevated atmospheric [CO_2_] and temperature. We observed that elevated [CO_2_] exerted more pronounced effects on the epidermis anatomy and leaf gas exchange, while air warming affected the leaf structure. When combined, these two environmental factors produced a set of anatomical adjustments that contributed to the acclimation of this species to future conditions increasing leaf biomass production, which is in agreement with other experimental [3] and productivity models [74]. Acclimation strategies were related to protection against the effects of a warmer environment and optimization of water use and carbon fixation, enhancing the performance of this species under these environmental conditions. Further studies should focus on the possible effects of elevated [CO_2_] and air warming on the flower, pollen, and ovule anatomy, and how these alterations influence the fitness of future generations.

## Supporting information

S1 Fig Meteorological conditions registered during the experimental period with *P*. *maximum* at Trop-T-FACE facility. (A) Average daily diurnal total solar radiation (*Rad*) and accumulated daily rainfall. (B) Average daily relative humidity (*Rh*) and average daily air temperature (*T*_*air*_). (C) Average daily soil temperature (*T*_*soil*_). *aT* plots = plots with ambient temperature; *eT* plots = warmed plots.
(TIF)

S2 Fig Chlorophyll fluorescence image of effective PSII quantum yield (Y[II]) measured at the end of the growing season in leaves of *P*. *maximum* at the Trop-T-FACE facility. [CO_2_] levels: *aC* (ambient [CO_2_], ~400 μmol mol^-1^) and *eC* (elevated [CO_2_], ~600 μmol mol^-1^). Temperature levels: *aT* (ambient temperature) and *eT* (2°C more than the ambient temperature). Means are followed by standard error (mean ± standard error). Relative values ranging from 0–1 of the Y(II) are displayed using an identical false color scale (bar is at the bottom of the image).
(TIF)

S3 Fig Malondialdehyde content (MDA) measured at the end of growing season in leaves of *P*. *maximum* at the Trop-T-FACE facility. Stack bars shows the standard error. [CO_2_] levels: *aC* (ambient [CO_2_], ~400 μmol mol^-1^) and *eC* (elevated [CO_2_], ~600 μmol mol^-1^). Temperature levels: *aT* (ambient temperature) and *eT* (2°C more than the ambient temperature). The ANOVA *p*-values are shown and significant effects (*p* < 0.05) are detailed in bold. [CO_2_] (isolated effect of elevated [CO_2_]), Temp. (isolated effect of air warming) and [CO_2_] × Temp. (interaction of elevated [CO_2_] × Temp.).
(TIF)
