# Correction: Increasing atmospheric CO_2_ and canopy temperature induces anatomical and physiological changes in leaves of the C_4_ forage species *Panicum maximum*

**DOI:** 10.1371/journal.pone.0238275

**Published:** 2020-08-20

**Authors:** Eduardo Habermann, Juca Abramo Barrera San Martin, Daniele Ribeiro Contin, Vitor Potenza Bossan, Anelize Barboza, Marcia Regina Braga, Milton Groppo, Carlos Alberto Martinez

## Notice of republication

At the time of the article’s publication, permissions were not obtained to offer the image in Fig 10 in *PLOS ONE* under the Creative Commons Attribution License. This article was republished on August 11, 2020, after the author obtained the necessary permission Please download this article again to view the correct version.

The caption for Fig 10 was erroneously included within the body of the text. This article was republished to correct this error and update the caption for Fig 10 to the following: “Created with BioRender. Elevated [CO_2_] (*eC*, green circle, isolated effect of CO_2_) exerted more pronounced effects on epidermis anatomy and leaf gas exchange. A CO_2_-enriched atmosphere reduced the differentiation of epidermal cells to stomata on both leaf surfaces, reducing stomatal density and index. In addition, stomatal aperture and transpiration were also decreased. Therefore, water use efficiency, photosynthesis and starch content increased. Due to low transpiration flux, soil water content was conserved during the experiment. Warming (*eT*, red circle, isolated effect of temperature) affected leaf structure and starch metabolism. Leaves developed protection mechanisms against the effects of a warmer environment with a thicker adaxial cuticle and reduced size of vascular bundles and bulliform cells. Under the combination of elevated [CO_2_] and warming (*eCeT*, purple circle, interaction of CO_2_ × temperature), warming cancelled the CO_2_ effect on soil water content and transpiration. However, when combined, these two environmental factors produced a set of anatomical adjustments that contributed to the acclimation of this species to future conditions increasing leaf biomass production. Down arrow: decrease. Up arrow: increase.”
