# Supplementary figures and images for: Increasing atmospheric CO2 and canopy temperature induces anatomical and physiological changes in leaves of the C4 forage species Panicum maximum
Source: PLoS One. 2019 Feb 19;14(2):e0212506. doi: 10.1371/journal.pone.0212506 (PMC6380572; doi:10.1371/journal.pone.0212506)

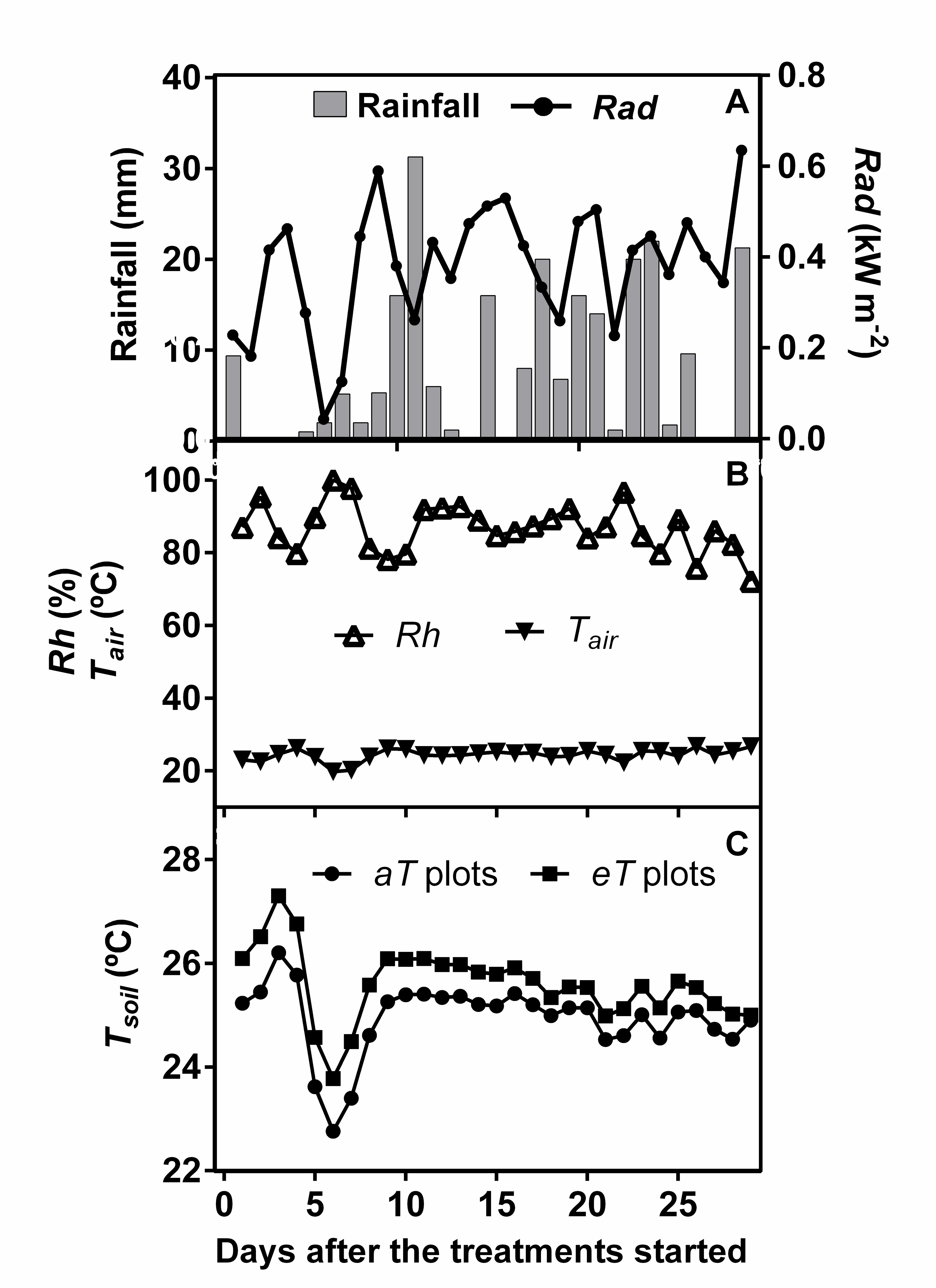

Supplement: S1 Fig — (A) Average daily diurnal total solar radiation (Rad) and accumulated daily rainfall. (B) Average daily relative humidity (Rh) and average daily air temperature (Tair). (C) Average daily soil temperature (Tsoil). aT plots = plots with ambient temperature; eT plots = warmed plots. (TIF) [file pone.0212506.s001.tif]

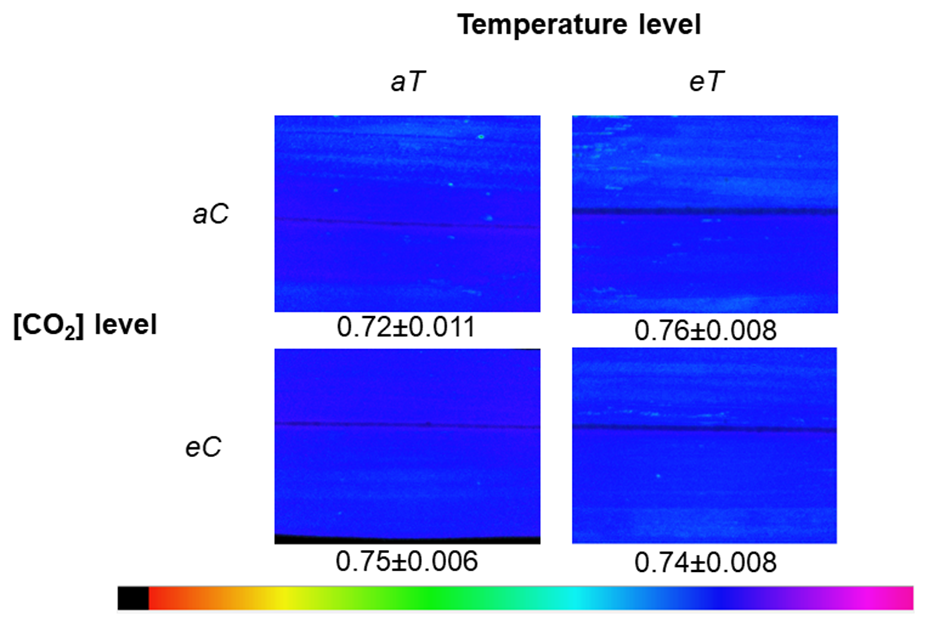

Supplement: S2 Fig — [CO2] levels: aC (ambient [CO2], ~400 μmol mol-1) and eC (elevated [CO2], ~600 μmol mol-1). Temperature levels: aT (ambient temperature) and eT (2°C more than the ambient temperature). Means are followed by standard error (mean ± standard error). Relative values ranging from 0–1 of the Y(II) are displayed using an identical false color scale (bar is at the bottom of the image). (TIF) [file pone.0212506.s002.tif]

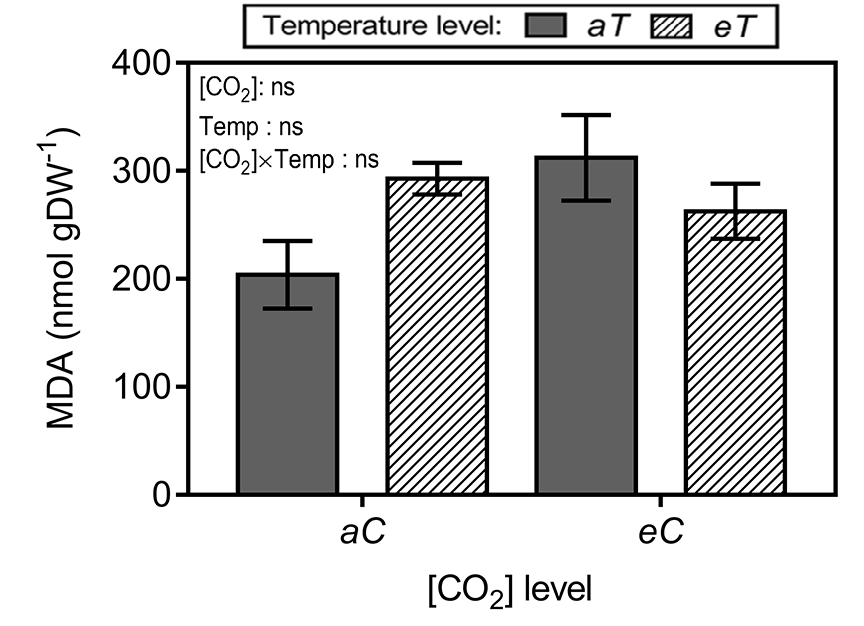

Supplement: S3 Fig — Stack bars shows the standard error. [CO2] levels: aC (ambient [CO2], ~400 μmol mol-1) and eC (elevated [CO2], ~600 μmol mol-1). Temperature levels: aT (ambient temperature) and eT (2°C more than the ambient temperature). The ANOVA p-values are shown and significant effects (p < 0.05) are detailed in bold. [CO2] (isolated effect of elevated [CO2]), Temp. (isolated effect of air warming) and [CO2] × Temp. (interaction of elevated [CO2] × Temp.). (TIF) [file pone.0212506.s003.tif]
